# Identification of high-risk plaque features in intracranial atherosclerosis: initial experience using a radiomic approach

**DOI:** 10.1007/s00330-018-5395-1

**Published:** 2018-04-09

**Authors:** Zhang Shi, Chengcheng Zhu, Andrew J. Degnan, Xia Tian, Jing Li, Luguang Chen, Xuefeng Zhang, Wenjia Peng, Chao Chen, Jianping Lu, Tao Jiang, David Saloner, Qi Liu

**Affiliations:** 1 Department of Radiology, Changhai Hospital, Second Military Medical University, Shanghai, 200433 China; 2 Department of Radiology and Biomedical Imaging, UCSF, San Francisco, CA USA; 3 Department of Radiology, The Children’s Hospital of Philadelphia, Philadelphia, PA USA

**Keywords:** Intracranial arteriosclerosis, Magnetic resonance imaging, Stroke, Atherosclerotic plaques, Basilar artery

## Abstract

**Objectives:**

To evaluate a quantitative radiomic approach based on high-resolution magnetic resonance imaging (HR-MRI) to differentiate acute/sub-acute symptomatic basilar artery plaque from asymptomatic plaque.

**Methods:**

Ninety-six patients with basilar artery stenosis underwent HR-MRI between January 2014 and December 2016. Patients were scanned with T1- and T2-weighted imaging, as well as T1 imaging following gadolinium-contrast injection (CE-T1). The stenosis value, plaque area/burden, lumen area, minimal luminal area (MLA), intraplaque haemorrhage (IPH), contrast enhancement ratio and 94 quantitative radiomic features were extracted and compared between acute/sub-acute and asymptomatic patients. Multi-variate logistic analysis and a random forest model were used to evaluate the diagnostic performance.

**Results:**

IPH, MLA and enhancement ratio were independently associated with acute/subacute symptoms. Radiomic features in T1 and CE-T1 images were associated with acute/subacute symptoms, but the features from T2 images were not. The combined IPH, MLA and enhancement ratio had an area under the curve (AUC) of 0.833 for identifying acute/sub-acute symptomatic plaques, and the combined T1 and CE-T1 radiomic approach had a significantly higher AUC of 0.936 (*p* = 0.01). Combining all features achieved an AUC of 0.974 and accuracy of 90.5%.

**Conclusions:**

Radiomic analysis of plaque texture on HR-MRI accurately distinguished between acutely symptomatic and asymptomatic basilar plaques.

**Key Points:**

*• High-resolution magnetic resonance imaging can assess basilar artery atherosclerotic plaque.*

*• Radiomic features in T1 and CE-T1 images are associated with acute symptoms.*

*• Radiomic analysis can accurately distinguish between acute symptomatic and asymptomatic plaque.*

*• The highest accuracy may be achieved by combining radiomic and conventional features.*

**Electronic supplementary material:**

The online version of this article (10.1007/s00330-018-5395-1) contains supplementary material, which is available to authorized users.

## Introduction

Increasing evidence supports the presentation of intracranial atherosclerotic disease (ICAD) as a major source of ischaemic cerebrovascular events worldwide, particularly in Asian, African American and Hispanic populations [1, 2]. The massive global health burden of ICAD is only partially understood despite increasing awareness including recent population investigations that implicate ICAD in dementia [3, 4]. Given the high risk of stroke recurrence in symptomatic patients with ICAD, accurate risk assessment of intracranial plaque is essential in guiding clinical management [1].

The traditional assessment of ICAD is by evaluation of luminal narrowing based on angiographic methods including: digital subtraction angiography (DSA), computed tomographic angiography (CTA) and magnetic resonance angiography (MRA) [5]. Due to the phenomenon of positive remodelling, in which an artery compensates for plaque growth by expansion of the outer wall, leaving the luminal diameter relatively unaffected, these angiographic methods have underestimated ICAD plaque burden, and an autopsy study attributed up to 27% of fatal ischaemic strokes to intracranial plaque with mild to moderate (30-75%) stenosis [6].

High-resolution magnetic resonance imaging (HR-MRI) of intracranial vessel walls facilitates the reliable, non-invasive characterisation of intracranial plaque, with clinical studies supporting its utility in ischaemic stroke risk estimation and ex vivo work validating its ability to classify plaque features [5, 7–13]. Posterior circulation plaques may be particularly underestimated by angiographic means because the basilar artery exhibits a propensity towards positive remodelling and the posterior circulation is predisposed to perforator occlusion [14–16]. Vertebrobasilar artery stenosis confers a significant risk of stroke, myocardial infarction and death [17]. Prior work supports the improved accuracy of HR-MRI over MRA in assessing basilar artery stenosis and its usefulness in guiding endovascular interventions [18–22].

Despite the favourable preliminary results, HR-MRI of intracranial plaque is complex and requires subjective assessment of plaque components. Advances in computing have enabled the development of automated, reproducible analysis methods, termed radiomics, to extract information from imaging studies to infer prognostic information for individual patients [23]. Radiomic approaches, such as texture analysis, show promise in multiple pathologies, including glioma, prostate cancer, hepatocellular carcinoma, and lung cancer, across different imaging modalities [24–27]. These radiomic approaches have not yet been applied to HR-MRI of ICAD.

This HR-MRI study of patients with ICAD including basilar artery plaque seeks to ascertain the ability of a radiomic approach including texture analysis to differentiate acute/sub-acute symptomatic plaque from asymptomatic plaque to serve as a framework for future prospective evaluations of basilar artery plaque risk.

## Materials and methods

### Study population

This study was approved by the Institutional Review Board of the Changhai Hospital (Shanghai, China), with all patients providing written informed consent. This study population was retrospectively selected from patients with intracranial stenosis who underwent intracranial HR-MRI between January 2014 and December 2016. All investigations were performed in accordance with the approved guidelines, including anonymisation of data during analysis. Patient inclusion criteria: (1) ischaemic stroke in basilar artery territory for the symptomatic group; (2) basilar artery stenosis on MRA or CTA; (3) ≥1 atherosclerotic risk-factors, including hypertension, diabetes mellitus, hypercholesterolaemia or cigarette smoking. Exclusion criteria: (1) non-atherosclerotic intracranial arterial disease, including vasculitis, moyamoya disease, dissection, reversible cerebral vasoconstriction syndrome and intracranial dolichoectasia; (2) chronic ischaemic stroke/transient ischaemic attack (TIA) (>12 weeks); (3) significant stenosis of the extracranial carotid arteries (> 30%) as assessed on ultrasound; (4) ascending aortic arch atheroma; (5) suspected cardio-embolic stroke; (6) known coagulopathy; (7) clinical contraindications to MRI, such as patients with pacemakers, certain metallic implants or severe claustrophobia.

### MRI acquisition

Patients were scanned using a 3-T whole-body MRI scanner (Skyra; Siemens Healthcare, Erlangen, Germany) with a 20-channel phased array head and neck coil. Patients were scanned using two dimensional high-resolution black blood T1- and T2- weighted fast-spin-echo sequences. T1 images were acquired both pre- and post-contrast (gadolinium). After an initial multi-plane localiser sequence, axial three-dimensional (3D) time-of-flight (TOF) MR angiography was performed to identify the location of the basilar stenosis. Then HR-MRI scanning was performed in planes perpendicular to the longitudinal orientation of the artery. Scan parameters: both T1- and T2-weighted sequences were acquired with 12 × 2-mm-thick slices; field of view 100 mm × 100 mm; matrix 256 × 320; in-plane resolution of 0.4 mm × 0.3 mm. T1 images had TR/TE = 581 ms/18 ms, echo train length (ETL) = 4 and number of excitations (NEX) = 4. T2 images had TR/TE = 2,890 ms/46 ms; ETL = 20; NEX = 3.

### Image analysis

All images were analysed by four radiologists (two radiology residents each with 3 years’ experience and two senior radiologists with 8 years’ experience in intracranial vessel wall imaging using these dedicated sequences). Each detected plaque was independently classified as acute/sub-acute symptomatic if a lesion was present on conventional neuroimaging (T2 FLAIR and DWI showed infarct) after an acute (<4 weeks) or sub-acute (4-12 weeks) ischaemic stroke/TIA with corresponding clinical symptoms. When multiple stenoses were present along the basilar artery, classification was based on the plaque at the location of maximal stenosis. A plaque was categorised as asymptomatic if detected in an asymptomatic patient and where conventional neuroimaging provided no evidence of infarct within the supplied vascular territory. Any disagreement was resolved by consensus.

Stenosis values were measured independently on HR-MRI images by one experienced radiologist (5 years’ experience in neuroradiology) [28]. Presence of fresh intraplaque haemorrhage (IPH) was identified if the signal intensity was >150% that of the nearby auricularis anterior muscles on pre-contrast T1-weighted images [29]. Lumen and outer wall boundaries were manually segmented on T2-weighted images at the slice with maximum plaque area using CMR Tools software (Cardiovascular Imaging Solutions, London, UK). Minimal luminal area (MLA) was computed based on the segmentation. MR signal intensity may vary within individuals due to factors such as coil positioning. Reproducibility of this area measurement method was previously reported to be excellent [30].

Gadolinium-based contrast agents were injected and post-contrast imaging was performed. The contrast enhancement ratio was measured at the slice of greatest enhancement, using adjacent grey matter (in a region of ~15 mm^2^ at the hippocampus) to normalise the signal intensity. The contrast enhancement percentage was calculated as { (signal of plaque [post-contrast]/signal of grey matter [post-contrast] ) / (signal of plaque [pre-contrast]/signal of grey matter[pre-contrast]) – 1} × 100%. Plaque burden (PB) was measured at the site of maximal stenosis, and was defined as (1 – lumen area/outer area) ×100%.

The radiomic features were analysed by an experienced reviewer (5 years’ experience in vessel wall imaging) using dedicated software (3D-Slicer) [31]. To evaluate the reproducibility of the radiomic analysis, another reviewer (10 years’ experience in vessel wall imaging) independently measured the radiomic features in a subset of patients (*n* = 40) who were randomly selected from the study population. Each reviewer segmented the plaque boundaries at the level of the largest plaque area on T1, T2 and CE-T1 images. For each sequence, 94 radiomic features—including intensity (maximal intensity, mean intensity, standard deviation of intensity, etc), shape-based features (area, length, etc.), and textures—were extracted and analysed according to a previous publication [32]. Textures include grey-level co-occurrence matrix (GLCM), grey-level run-length matrix (GLRLM) and grey-level size-zone matrix (GLSZM). The textural features were described patterns or the spatial distribution of voxel intensities, which were calculated respectively from grey level co-occurrence (GLCM) and grey-level run-length (GLRLM) texture matrices. Determining texture matrix representations requires the voxel intensity values within the volume of interest (VOI) to be discretised. This discretisation step not only reduces image noise but also normalises intensities across all patients, allowing for a direct comparison of all calculated textural features between patients. The detailed definition of these features can be found in the previous publication [32]. The reproducibility of radiomic feature quantification was calculated by comparing the results from the two reviewers.

### Statistical analysis

All statistical analyses were performed using MATLAB (R2013a; The Mathworks, Natick, MA, USA) and SPSS24.0. The mean and standard deviation (SD) were recorded for continuous variables, and frequency and percentage were recorded for categorical variables. For each variable, a normality test was performed. Univariate analysis was first performed to assess the association between each variable and acute symptomatic status. As all variables obtained from the radiomic analysis exhibited normal distributions, *t*-tests were used for the comparison of continuous variables. Chi-squared tests were used for the categorical variables. Multivariate analysis was then performed which included the variables that had *p* < 0.10 in the univariate tests. The odds ratios (ORs) with 95% confidence intervals (CIs) were calculated by a logistic regression model with stepwise selection of variables. Additionally, *t*-tests were used to first select the features with *p* < 0.05 in each sequence, and the features with significant differences and area under curve (AUC) values >0.65 were set as inputs for the random forest training features. To evaluate the diagnostic performance of radiomic features, supervised machine-learning methods using random forests [33] were applied to classify acute symptomatic from asymptomatic plaques.

A random forest is a meta-estimator that fits a number of decision-tree classifiers on various sub-samples of the dataset; ten classification trees with three layers were applied in this model; after each tree was voted the decision for classification result, the result was averaged to improve the predictive accuracy and control over-fitting. In random forests, each tree in the ensemble is built from a sample drawn with replacement from the training set. In addition, when splitting a node during the construction of the tree, the split that is picked is the best split among a random subset of the features.

The diagnostic performance was described using receiver operating characteristic (ROC) curves and AUC values. ROC curves were compared using the method developed by DeLong et al. [34] Reproducibility was evaluated by Bland-Altman plots and intraclass coefficient (ICC) using a two-way random model with absolute measurements. A *p* value <0.05 was considered statistically significant.

## Results

### Patients

A total of 174 patients met the inclusion criteria. Seventy-eight patients were excluded due to intracranial aneurysm (*n* = 51), moyamoya disease (*n* =1 3), vasculitis (*n* = 7), dissection (*n* = 5) and bad image quality (*n* = 2). As a result, 96 patients were included in the final analysis. The demographic data of the patients is shown in Table 1. There were 43 acute, 18 sub-acute and 35 asymptomatic patients.

**Table 1:** Patient demographic data

Characteristics	*n*/total (%)
Gender	
Male	64 (66.67)
Female	32 (33.33)
Age^a^	61.85 ± 10.08
Diabetes mellitus	34 (35.05)
Hypertension	78 (80.41)
Hyperlipidaemia	49 (50.52)
Smoking	27 (27.84)
Stenosis (>50%)	60 (61.86)
Clinical symptom	
Acute symptomatic	43 (44.79)
Sub-acute symptomatic	18 (18.75)
Asymptomatic	35 (36.46)

^a^Mean (± SD)

### Traditional assessment of the intracranial atherosclerotic plaques

**Fig. 1:**
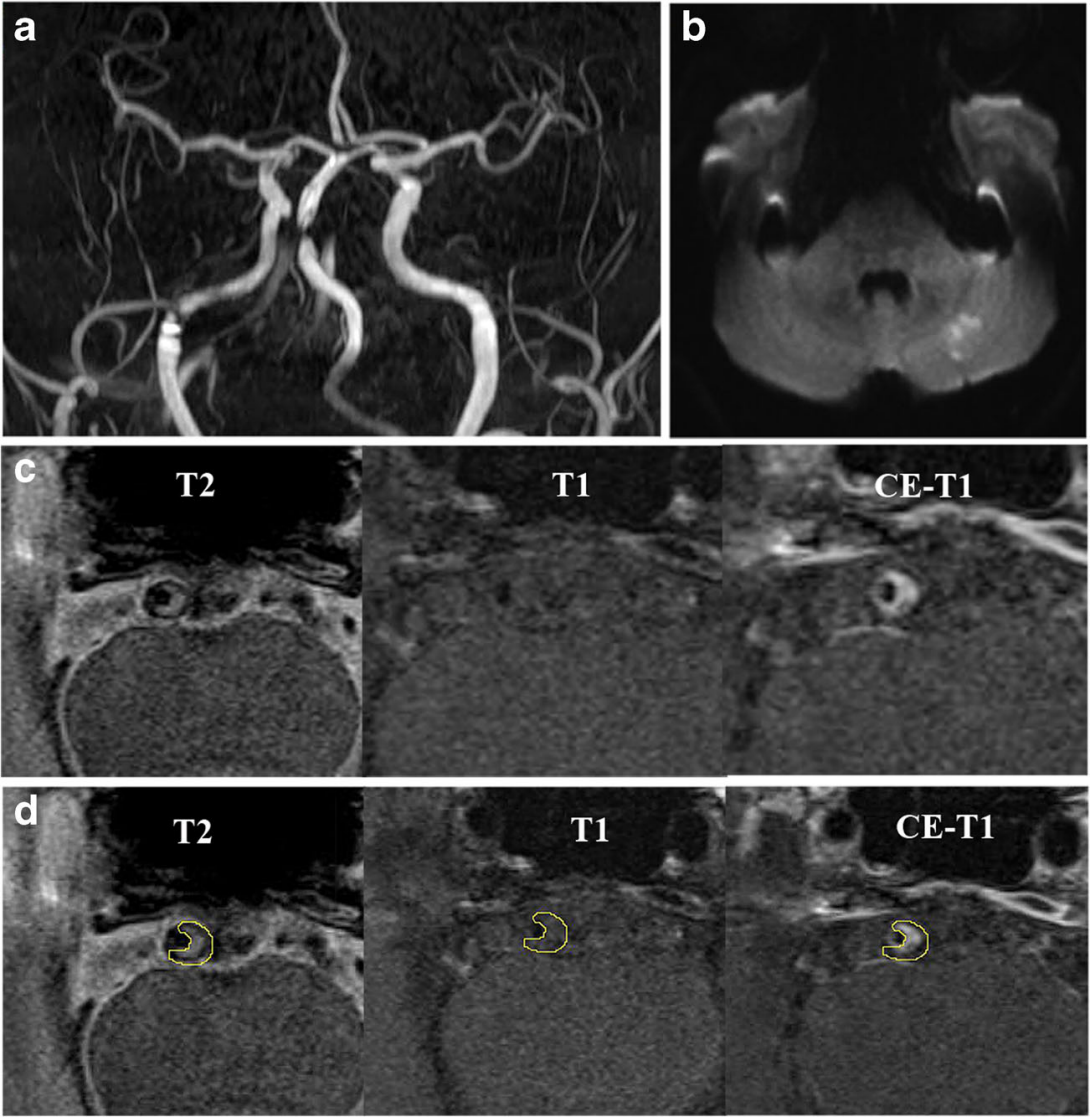
MRI images showing BA atherosclerotic plaque in a symptomatic patient. TOF-MRA (**a**) demonstrates stenosis, and DWI (**b**) shows the acute infarcts which are scattered and patchy in distribution within the left cerebellum. T2-weighted, T1-weighted and CE-T1-weighted images (*from left to right*) in the middle slice of the BA plaque are shown in **c** and **d**

**Fig. 2:**
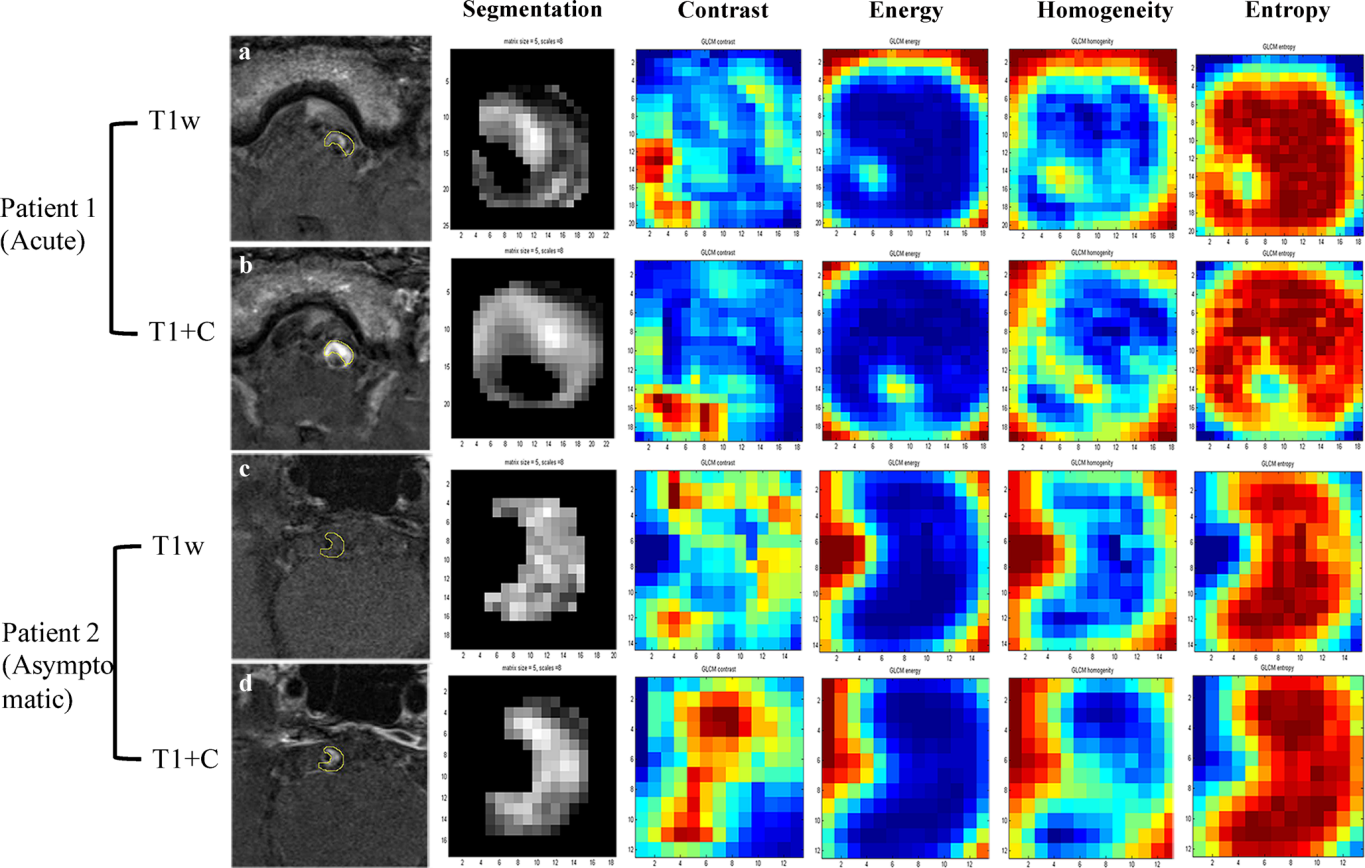
Radiomics analysis in two sample patients. Patient 1 is a 63-year-old man with acute stroke on the stem. Patient 2 is a 57-year-old man free of symptoms. T1-weighted images are shown in **a** and **c**, and CE-T1 images are shown in **b** and **d**. Four representative GLCM radiomics features from 94 features are shown (contrast, energy, homogeneity, entropy)

**Fig. 3:**
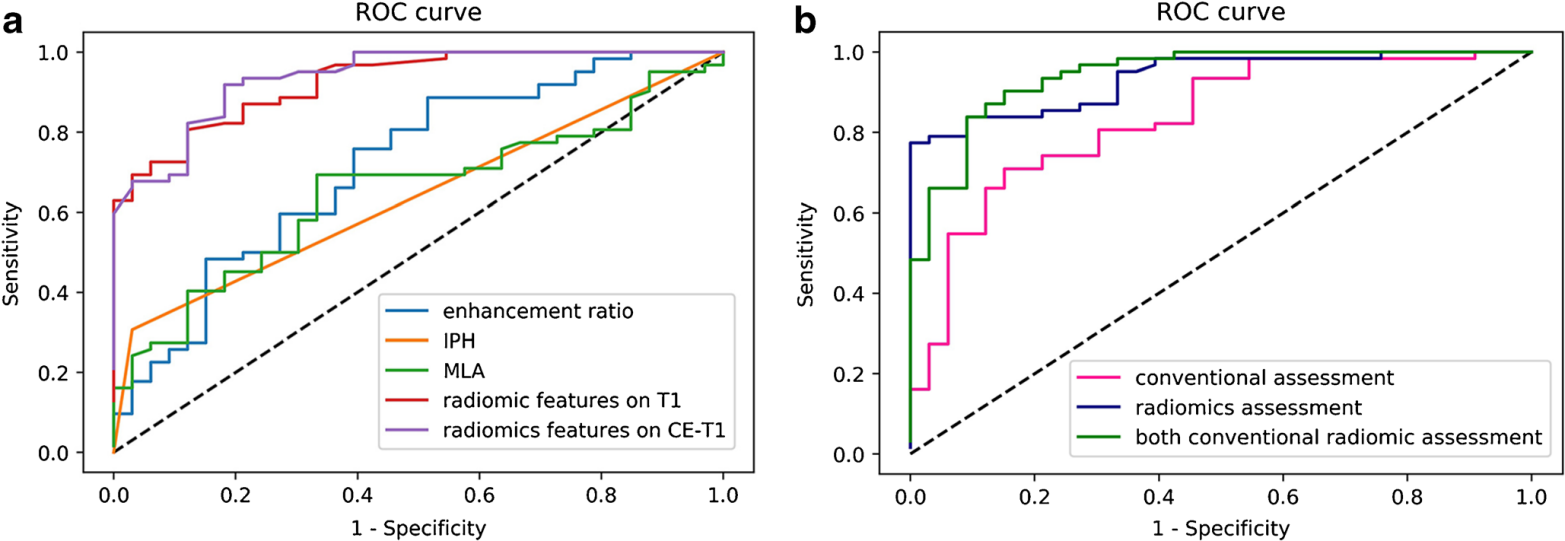
ROC curves to differentiate acute/sub-acute symptomatic and asymptomatic plaques. The curves on the left (**a**) show the diagnostic performance of each independent parameter. The curves on the right (**b**) shows diagnostic performance of the combined traditional/radiomics model and a model combination of all features. Radiomic features had significantly higher AUC values compared with traditional features (*p* = 0.01)

Clinical and radiological characteristics of the intracranial atherosclerotic plaques are summarised in Table 2. Sample patient images are shown in Figs. 1 and 2. Univariate analysis showed that gender, smoking, IPH, MLA and enhancement ratio were associated with acute/sub-acute symptomatic plaques (*p* < 0.1). Multivariate logistic regression analysis showed that IPH [odds ratio (OR) = 17.803; 95% CI, 2.093-151.472], MLA (OR = 1.515; 95% CI, 1.123-2.043) and enhancement ratio (OR = 71.979; 95% CI, 3.840-1349.211) were independent predictors of acute/sub-acute symptoms, and the AUC of the ROC curves was 0.638, 0.650 and 0.717, respectively (Fig. 3). The AUC value was improved to 0.833 when combining IPH, MLA and enhancement ratio in the model, with an optimised sensitivity of 84.8%, specificity of 71.0% and accuracy of 74.7%. Details of the diagnostic performance data are listed in Table 4.

**Table 2:** Clinical and radiological features of the intracranial atherosclerotic plaques

	Intracranial atherosclerosis			Multivariate odds ratio (95 %CI) ^b^
	Acute/sub-acute symptomatic	Asymptomatic	*p* value^a^	
Gender			0.023	
Male	46	18		
Female	15	17		
Age	61.68 ± 10.75	62.14 ± 8.92	0.828	
Diabetes mellitus^c^	23	11	0.716	
Hypertension^d^	48	30	0.323	
Hyperlipidaemia^d^	23	11	0.574	
Smoking	23	4	0.013	
IPH	19	1	0.003	17.803 (2.093, 151.472) *p* = 0.008
Plaque burden (%)	83.24 ± 9.71	85.04 ± 7.51	0.345	
MLA (mm^3^)	3.78 ± 2.80	2.39 ± 1.46	0.008	1.515 (1.123, 2.043) *p* = 0.007
Degree of stenosis (%)	53.69 ± 15.19	53.76 ± 16.73	0.984	
Enhancement ratio (%)	24.20 ± 29.46	3.38 ± 21.91	<0.001	71.979 (3.840, 1349.211) *p* = 0.004

*IPH* intraplaque haemorrhage, *MLA* minimum luminal area

^a^Two independent-samples *t*-test for continuous variables

^b^Results from multivariate analysis

^c^Diabetes mellitus: two fasting glucose measurements above 126 mg/dl (7.0 mmol/l) or glycated haemoglobin (HbA1C) ≥48 mmol/mol

^d^Hypertension: a systolic or a diastolic blood pressure measurement consistently higher than an accepted normal value (this is above 139 mmHg systolic, 89 mmHg diastolic)

^e^Hyperlipidemia: abnormally elevated levels of any or all lipids or lipoproteins in the blood;LDL-C >130mg/dl, HDL-C <40mg/dl, TG >150mg/dl, TC > 200mg/dl

### Radiomic assessment of intracranial atherosclerotic plaques

Samples of the radiomic features are shown in Fig. 2. Random forest analysis showed seven radiomic features in T1 images and three features in CE-T1 images that were independently associated with acute/sub-acute symptoms (Table 3), while none of the features in T2 images were statistically significant. Diagnostic performance data of radiomics are listed in Table 4. T1 radiomic features had an AUC value of 0.893 and CE-T1 radiomic features, 0.918. When combining all the radiomic features from T1 and CE-T1 images, the AUC value improved to 0.936, with an optimised sensitivity of 97.0% and specificity of 79.0%, and accuracy of 83.2% (Fig. 3). Radiomic features had significantly higher AUC values than traditional features (*p* = 0.01).

**Table 3:** Independent radiomics features on T1 and CE-T1 images

	Acute/sub-acute symptomatic	Asymptomatic	*p* value
Features on T1 images			
shape_Maximum3DDiameter	5.16 ± 1.41	4.25 ± 0.95	<0.001
shape_Maximum2DDiameterSlice	5.21 ± 1.42	4.27 ± 0.98	<0.001
shape_Volume	40.16 ± 24.01	27.41 ± 14.63	0.005
shape_SurfaceArea	71.83 ± 29.23	53.43 ± 19.58	0.001
shape_Maximum2DDiameterColumn	4.40 ± 1.41	3.61 ± 0.91	0.004
glcm_Entropy	5.78 ± 0.75	5.36 ± 0.73	0.011
glrlm_RunLengthNonUniformity	102.89 ± 62.18	37.03 ± 16.46	0.006
Features on CE-T1 images			
firstorder_Uniformity	0.07 ± 0.03	0.09 ± 0.02	0.005
glcm_MaximumProbability	0.04 ± 0.02	0.06 ± 0.05	0.004
glcm_Entropy	6.01 ± 0.84	5.43 ± 1.21	0.007

Definitions of the radiomics features can be found in the reference: https://www.ncbi.nlm.nih.gov/pubmed/24892406

**Table 4:** The diagnostic accuracy findings

	DA	AUC	sensitivity	specificity	LR+	1/LR-
Traditional assessment	0.747	0.833	0.848	0.71	2.926	4.686
Radiomics (T1-weighted)	0.8	0.893	0.909	0.726	3.318	7.986
Radiomics (CE-T1)	0.819	0.918	0.909	0.823	5.136	9.053
All of radiomics	0.832	0.936	0.97	0.79	4.619	26.33
Traditional & radiomics	0.905	0.974	0.939	0.871	7.279	14.28

*DA* diagnostic accuracy, *AUC* area under the curve, *LR+* positive likelihood ratio, *LR-* negative likelihood ratio

When combining all radiomic features and IPH/MLA/enhancement ratio, the AUC value was improved to 0.974; however, it was not significantly higher than radiomic features alone (*p* = 0.275). The optimised sensitivity was 93.9%, the specificity was 87.1% and the accuracy was 90.5%.

### Reproducibility of traditional and radiomic measurements

Bland-Altman plots of traditional and radiomic measurements and the ICC values are shown in Supplementary Figs. 1 and 2. The ICCs of the traditional features for the two reviewers in measuring the degree of stenosis, PB, MLA and enhancement ratio were 0.782, 0.778, 0.875, 0.792 respectively (average ICC, 0.806). The Cohen’s kappa for the two reviewers in determining IPH was 0.811.

The average ICCs for the two reviewers in measuring the ten independent radiomic features were 0.836 (range, 0.798-0.881) for T1 images and 0.837 (range, 0.806-0.858) for CE-T1 images.

## Discussion

Quantitative radiomic analysis accurately differentiated symptomatic from asymptomatic basilar artery atherosclerotic plaques on HR-MRI, outperforming conventional imaging variables and clinical risk factors. As there is now increasing recognition that ICAD is more common than previously thought, with an estimated prevalence of ICAD in the United States of 34% [10], it is apparent that characterisation of intracranial plaque must go beyond assessing the presence of plaque or the degree of stenosis to accurately determine the risk of future cerebrovascular event. In this study, radiomic analysis accurately assigned symptom status to basilar artery plaque with an excellent AUC of 0.936, while clinical imaging features alone had a moderate AUC of 0.833. This retrospective study suggests radiomic analysis could substantially improve assessment of acute symptomatic versus chronic/asymptomatic basilar intracranial atherosclerosis.

Qualitative features of intracranial plaque such as plaque enhancement, suggesting active inflammation, or T_1_-weighted hyperintense signal, indicating intraplaque haemorrhage, have been associated with symptomatic, culprit plaque [11, 35]. In addition, quantitative plaque features such as stenosis, plaque area and enhancement ratio may also be helpful. However, both qualitative and quantitative imaging features are limited by variable observer expertise, limited reproducibility and heterogeneous imaging protocols. A key advantage of the radiomic method is that it can provide reader-independent findings [23]. These radiomic features cannot be readily discerned by a human observer and may substantially add to the diagnostic confidence and accuracy of a study in ways that a human interpreter is not capable of appreciating. In our analysis, the highest AUC (0.974) was found using radiomic basilar artery plaque features in conjunction with an assessment of MLA, the presence of intraplaque haemorrhage and gadolinium enhancement.

Compared with conventional methods, radiomic analysis is more quantitative and may detect “agnostic” features that the radiologist cannot appreciate, such as uniformity or randomness (entropy) of the intensities on the image. Radiomics provides richer information about intensity, shape, size or volume, and texture of the plaque/tumour. On the other hand, conventional measurements only provide limited information about image features. For example, IPH has been studied as a potential marker of high-risk intracranial plaque, but previous studies commonly used a binary categorisation (present or not) [29]. The quantitative signal intensity, volume/shape and the complicated distribution of IPH (focal or diffuse, close to the lumen or close to the wall) have rarely been studied, because routine image analysis by clinical radiologists does not yield this level of assessment. A quantitative radiomics matrix contains detailed information.

Because of these advantages, radiomics has shown great prognostic value. For instance, a study by Aerts et al. [32] showed that a prognostic radiomic signature, capturing intratumour heterogeneity, was associated with underlying gene-expression patterns in lung and head-and-neck cancer patients. These data suggest that radiomics identifies prognostic phenotypes existing in both lung and head-and-neck cancer. In a study of 282 patients with early-stage non-small cell lung cancer, radiomics showed additional value other than clinical-pathological risk factors for estimation of disease-free survival time [36].

We found the independent radiomics features from T1 and CE-T1 images were different. This is because the signal characteristics in pre- and post-contrast T1-weighted images reflect different pathophysiological features of plaque. For example, the high signal on pre-contrast T1-weighted images is possibly intraplaque haemorrhage (IPH), and the low signal is thought to represent the lipid core (as described in an ex vivo study [13]); on the other hand, the high signal on post-contrast T1-weighted images is attributed to the contrast uptake by fibrous cap or possible vasa vasorum.

In our study, the random forest method has been chosen. As a result of this randomness, the bias of the forest usually slightly increases (with respect to the bias of a single non-random tree) but, due to averaging, its variance also decreases, usually more than compensating for this increase in bias, yielding a better overall model. This procedure leads to better model performance because it decreases the variance of the model, without increasing the bias. This means that while the predictions of a single tree are highly sensitive to noise in its training set, the average of many trees is not, as long as the trees are not correlated. Simply training many trees on a single training set would give strongly correlated trees (or even the same tree many times, if the training algorithm is deterministic); bootstrap sampling is a way of de-correlating the trees by showing them different training sets.

To our knowledge, this study is the first investigation of ICAD with HR-MRI using a radiomic analysis. An ultrasound study of extra-cranial carotid artery atherosclerotic plaque applied texture feature analysis to classify plaque echogenicity with success [37]. Those authors achieved an accuracy of 88% in identifying anechoic, intermediate, and hyperechoic plaques with an AUC of 0.918 for the anechoic plaque, which is thought to confer an increased risk of cardiovascular and cerebrovascular events [37]. Another group used principal component analysis of carotid plaque texture and echogenicity to determine variables associated with plaque instability on histology that were independent of stenosis [38]. Texture analysis methods have also been applied to carotid ultrasound elastography using HR-MRI as a reference to improve plaque vulnerability assessment [39]. These ultrasound-based studies of extracranial atherosclerosis support the use of radiomic analysis to reveal quantitative variables within images to improve risk estimates associated with atherosclerotic plaque beyond plaque size or qualitative features. This study similarly extends radiomic analysis of intracranial plaque by classifying intracranial basilar artery plaque into acute/sub-acute symptomatic versus asymptomatic based on quantitative information extracted from HR-MRI.

Measurements that include plaque burden and minimum luminal area as determined on HR-MRI have substantially improved the assessment of culprit plaques compared with degree of stenosis alone in a study of middle cerebral artery atherosclerosis [40]. However, the diagnostic performance of these variables was suboptimal, with the combination of plaque burden, minimum luminal area and stenosis together resulting in an accuracy of 71.5% [40], which is comparable to the accuracy reported in our study when using enhancement ratio, MLA and IPH (74.7%). Radiomic analysis has the benefit of identifying quantitative variables within imaging data to improve accuracy (83.2% as shown in this study) and diagnostic confidence beyond conventional measurements. The favourable accuracy values in this study over those previously reported by conventional HR-MRI support the use of radiomic analysis to improve identification of acute symptomatic plaque.

It is hoped that radiomic approaches applied to HR-MRI of the basilar artery will result in improved accuracy of stroke risk assessment, which is especially important given the high-risk nature of symptomatic basilar artery ICAD [17]. This risk assessment could then be used to initiate more effective secondary prevention strategies by more appropriately assigning high-risk patients with posterior circulation ischaemic events due to non-stenotic plaque to more aggressive dual antiplatelet therapy. Before this radiomic plaque information can be applied more broadly, prospective treatment studies are needed to investigate the role of aggressive medical management in patients with non-stenotic, high-risk plaque as current treatment recommendations are based on prior investigations showing the benefit of dual antiplatelet therapy such as SAMMPRIS that was restricted to patients with high-grade stenosis of 70-99% occlusion [41, 42]. Others have suggested a possible role for endovascular therapy of selected patients with vertebrobasilar stenosis, and basilar artery plaque radiomic assessment may assist in risk assessment and treatment decision-making. However, more data are needed regarding the role endovascular therapy in these conditions [43].

### Limitations

Although this study provided significant results, the sample size used to train the texture analysis of plaque was relatively small (*n* = 96). Future work would benefit from training with a larger data set. In addition, our analysis was performed using two-dimensional (2D) imaging data. It is possible that the use of 3D imaging acquisitions could better characterise plaque features for improved radiomic analysis. Also, reliance on a 2D TOF sequence to initially identify plaque may have underestimated the presence of non-stenotic atherosclerotic plaque. Three-dimensional imaging methods (up to 0.5 mm isotropic) [44] can reduce the partial volume effect of 2D acquisition and allow the detection of non-stenotic basilar artery or PCA plaques. However, the long scan time and wider point spread function induced by long echo trains must also be considered [45, 46]. Another potential improvement is the use of automated segmentation. Manual segmentation of the small intracranial plaque is still a challenging task. Automatic segmentation would reduce inter-observer variability and improve the feasibility of applying these methods to larger datasets.

This retrospective study was not able to ascertain the ability of radiomic analysis to predict the risk of future stroke risk, which is of paramount importance. Future prospective studies associating plaque features with long-term stroke risk through radiomic techniques could substantially shift the imaging paradigm from simple assessment of stenosis and subjective plaque components to a rigorous evaluation of objective and quantifiable plaque characteristics.

As a nascent field, radiomic methods may require additional refinement to improve accuracy. Diagnostic accuracy and predictive power are dependent on the quality of the data set utilised to train radiomic algorithms [23]. Leveraging quantitative imaging data with large population studies could provide actionable information to generate robust automated assessments of stroke risk based on vessel wall MRI of ICAD.

### Conclusions

Radiomic analysis of basilar artery plaque texture on HR-MRI accurately distinguished between acute/sub-acute symptomatic and asymptomatic plaque. Radiomic features alone successfully predicted plaque symptom status over clinical imaging features in this retrospective study. Prospective studies are needed to further ascertain the ability of radiomic analysis of intracranial plaque HR-MRI to predict future stroke risk.

## Electronic supplementary material

ESM 1 (PDF 67 kb)

ESM 2 (GIF 142 kb)

High resolution image (TIFF 7376 kb)

ESM 3 (GIF 134 kb)

High resolution image (TIFF 26585 kb)

## Abbreviations

BA: Basilar artery
GLCM: Grey-level co-occurrence matrix
GLRLM: Grey-level run-length matrix
GLSZM: Grey-level size-zone matrix
HR-MRI: High-resolution magnetic resonance imaging
ICAD: Intracranial atherosclerotic disease
ICC: Intraclass coefficient
IPH: Intraplaque haemorrhage
MLA: Minimal luminal area
PB: Plaque burden
