# Supplementary material for: Identification of high-risk plaque features in intracranial atherosclerosis: initial experience using a radiomic approach
Source: Eur Radiol. 2018 Apr 9;28(9):3912–21. doi: 10.1007/s00330-018-5395-1 (PMC6081255; doi:10.1007/s00330-018-5395-1)
Supplement: Supplementary file 1 — (PDF 67 kb) [file 330_2018_5395_MOESM1_ESM.pdf]

All of the Radiomic Features from T1 and CE-T1 images

Features on T1 images	Acute/sub-acute symptomatic	Asymptomatic	p-value
original_shape_Volume	40.16±24.01	27.41±12.63	0.005
original_shape_SurfaceVolumeRatio	1.93±0.30	2.07±0.37	0.048
original_shape_SurfaceArea	71.83±29.23	53.43±19.58	0.001
original_shape_Sphericity	0.78±0.07	0.82±0.07	0.009
original_shape_SphericalDisproportion	1.29±0.11	1.23±0.12	0.016
original_shape_MinorAxis	4.22±1.21	3.45±0.99	0.002
original_shape_Maximum3DDiameter	5.16±1.41	4.25±0.95	<0.001
original_shape_Maximum2DDiameterSlice	5.16±1.41	4.25±0.95	<0.001
original_shape_Maximum2DDiameterRow	4.02±1.28	3.32±1.11	0.009
original_shape_Maximum2DDiameterColumn	4.40±1.41	3.60±0.91	0.004
original_shape_MajorAxis	5.73±1.42	4.85±0.98	0.002
original_shape_Elongation	0.74±0.13	0.71±0.13	0.297
original_shape_Compactness2	0.48±0.12	0.56±0.14	0.005
original_shape_Compactness1	0.04±0.004	0.04±0.005	0.009
original_glszm_SmallAreaHighGrayLevelEmphasis	118.63±79.21	85.03±31.08	0.02
original_glszm_GrayLevelVariance	20.53±16.40	18.50±7.10	0.494
original_glszm_GrayLevelNonUniformityNormalized	0.09±0.01	0.09±0.02	0.481
original_glrlm_ShortRunLowGrayLevelEmphasis	0.05±0.04	0.08±0.05	0.009
original_glrlm_ShortRunHighGrayLevelEmphasis	131.60±83.59	91.78±31.15	0.06
original_glrlm_ShortRunEmphasis	0.95±0.02	0.96±0.02	0.141
original_glrlm_RunVariance	0.07±0.04	0.06±0.03	0.054
original_glrlm_RunPercentage	0.94±0.02	0.95±0.02	0.062
original_glrlm_RunLengthNonUniformityNormalized	0.88±0.04	0.89±0.05	0.141
original_glrlm_RunLengthNonUniformity	53.08±30.90	37.03±16.46	0.006
original_glrlm_RunEntropy	3.90±0.38	3.77±0.27	0.082
original_glrlm_LowGrayLevelRunEmphasis	0.05±0.04	0.08±0.06	0.013
original_glrlm_LongRunLowGrayLevelEmphasis	0.06±0.05	0.09±0.07	0.056
original_glrlm_LongRunHighGrayLevelEmphasis	161.73±93.82	110.19±35.20	0.093
original_glrlm_LongRunEmphasis	1.21±0.10	1.18±0.08	0.075
original_glrlm_HighGrayLevelRunEmphasis	137.18±85.46	95.28±31.70	0.007
original_glrlm_GrayLevelVariance	19.15±16.15	17.19±7.16	0.505
original_glrlm_GrayLevelNonUniformityNormalized	0.09±0.01	0.09±0.02	0.635
original_glrlm_GrayLevelNonUniformity	5.11±2.61	3.75±1.39	0.006
original_gldm_SmallDependenceLowGrayLevelEmphasis	0.08±0.06	0.06±0.04	0.002
original_gldm_SmallDependenceHighGrayLevelEmphasis	100.76±71.73	72.79±30.39	0.033
original_gldm_SmallDependenceEmphasis	0.70±0.09	0.73±0.11	0.103
original_gldm_LowGrayLevelEmphasis	0.05±0.04	0.08±0.05	0.016
original_gldm_LargeDependenceLowGrayLevelEmphasis	0.12±0.18	0.14±0.15	0.531
original_gldm_LargeDependenceHighGrayLevelEmphasis	337.69±176.15	219.43±97.73	0.001
original_gldm_LargeDependenceEmphasis	2.78±0.88	2.49±0.71	0.106
original_gldm_HighGrayLevelEmphasis	136.75±85.14	94.83±31.65	0.007
original_gldm_GrayLevelVariance	18.74±16.09	16.79±7.20	0.505
original_gldm_GrayLevelNonUniformityNormalized	0.095±0.027	0.097±0.017	0.77

original_gldm_GrayLevelNonUniformity	5.65±3.02	4.09±1.59	0.006
original_gldm_DependenceVariance	0.49±0.31	0.41±0.20	0.176
original_gldm_DependenceNonUniformityNormalized	0.49±0.10	0.54±0.15	0.078
original_gldm_DependenceNonUniformity	31.16±17.90	22.54±10.17	0.011
original_gldm_DependenceEntropy	4.40±0.43	4.16±0.36	0.006
original_glcm_SumSquares	18.57±16.79	16.19±6.65	0.429
original_glcm_SumEntropy	3.96±0.52	3.70±0.49	0.019
original_glcm_SumAverage	21.04±6.38	17.50±3.51	0.004
original_glcm_MaximumProbability	0.05±0.01	0.05±0.02	0.358
original_glcm_JointEnergy	0.02±0.01	0.03±0.02	0.051
original_glcm_JointAverage	10.52±3.19	8.75±1.75	0.004
original_glcm_InverseVariance	0.24±0.05	0.22±0.05	0.062
original_glcm_Imc2	0.96±0.04	0.98±0.02	0.086
original_glcm_Imc1	0.41±0.11	0.48±0.14	0.009
original_glcm_Idn	0.84±0.02	0.83±0.03	0.006
original_glcm_Idmn	0.94±0.02	0.93±0.02	0.011
original_glcm_Idm	0.23±0.05	0.21±0.05	0.08
original_glcm_Id	0.32±0.04	0.31±0.05	0.114
original_glcm_Homogeneity2	0.23±0.04	0.21±0.05	0.08
original_glcm_Homogeneity1	0.23±0.05	0.31±0.05	0.123
original_glcm_Entropy	5.78±0.75	5.36±0.73	0.011
original_glcm_Dissimilarity	4.05±1.22	4.16±0.91	0.665
original_glcm_DifferenceVariance	11.31±10.18	9.27±2.97	0.257
original_glcm_DifferenceEntropy	3.15±0.36	2.98±0.25	0.018
original_glcm_DifferenceAverage	4.05±1.22	4.16±0.91	0.665
original_glcm_Correlation	0.14±0.15	0.12±0.16	0.52
original_glcm_Contrast	29.42±22.88	27.75±11.62	0.691
original_glcm_ClusterTendency	44.87±46.32	36.99±18.37	0.344
original_glcm_ClusterShade	109.07±568.19	58.35±223.96	0.619
original_glcm_ClusterProminence	13439.79±36096.56	5184.10±7161.47	0.191
original_glcm_Autocorrelation	124.54±80.43	81.96±30.55	0.004
original_firstorder_Variance	11678.48±10137.24	10598.34±4828.87	0.56
original_firstorder_Uniformity	0.095±0.027	0.097±0.017	0.674
original_firstorder_TotalEnergy	6766448.88±7067367.51	4665427.94±4056723.69	0.114
original_firstorder_StandardDeviation	101.77±36.66	100.71±21.70	0.877
original_firstorder_Skewness	0.01±0.50	0.28±0.52	0.016
original_firstorder_RootMeanSquared	373.17±141.19	393.25±124.94	0.49
original_firstorder_RobustMeanAbsoluteDeviation	53.45±18.75	55.56±13.15	0.561
original_firstorder_Range	486.76±191.28	435.85±93.76	0.149
original_firstorder_Minimum	121.19±102.51	184.47±127.10	0.009
original_firstorder_Median	356.40±138.84	375.94±124.99	0.497
original_firstorder_MeanAbsoluteDeviation	79.93±27.91	81.13±17.83	0.822
original_firstorder_Mean	358.07±138.87	378.85±127.08	0.472
original_firstorder_Maximum	607.95±234.24	620.32±155.90	0.783
original_firstorder_Kurtosis	3.22±0.81	2.94±1.18	0.18

original_firstorder_InterquartileRange	127.42±45.70	136.07±42.91	0.367
original_firstorder_Entropy	3.68±0.42	3.58±0.25	0.194
original_firstorder_Energy	10826270.2±11307718.9	7490880.68±6470953.83	0.117
original_firstorder_90Percentile	484.62±173.99	510.12±144.34	0.469
original_firstorder_10Percentile	234.01±112.19	257.39±121.13	0.345
general_info_VoxelNum	64.26±38.42	44.12±19.98	0.005

Features on CE-T1 images	Acute/sub-acute symptomatic	Asymptomatic	p-value
original_shape_Volume	39.55±21.29	31.56±22.57	0.093
original_shape_SurfaceVolumeRatio	1.93±0.32	2.08±0.56	0.087
original_shape_SurfaceArea	70.73±27.44	56.55±28.12	0.021
original_shape_Sphericity	0.78±0.07	0.82±0.07	0.011
original_shape_SphericalDisproportion	1.29±0.11	1.23±0.11	0.012
original_shape_MinorAxis	4.25±1.22	3.56±1.34	0.012
original_shape_Maximum3DDiameter	5.05±1.25	4.91±3.38	0.772
original_shape_Maximum2DDiameterSlice	5.05±1.25	4.38±1.38	0.019
original_shape_Maximum2DDiameterRow	3.99±1.27	3.50±1.51	0.096
original_shape_Maximum2DDiameterColumn	4.40±1.26	3.61±1.37	0.006
original_shape_MajorAxis	5.58±1.23	5.25±2.43	0.382
original_shape_Elongation	0.76±0.14	0.70±0.17	0.068
original_shape_Compactness2	0.49±0.13	0.57±0.14	0.008
original_shape_Compactness1	0.036±0.004	0.039±0.005	0.011
original_glszm_SmallAreaHighGrayLevelEmphasis	249.20±256.14	150.77±95.24	0.036
original_glszm_GrayLevelVariance	57.79±70.30	29.33±16.53	0.024
original_glszm_GrayLevelNonUniformityNormalized	0.07±0.02	0.08±0.03	0.003
original_glrlm_ShortRunLowGrayLevelEmphasis	0.04±0.03	0.05±0.03	0.079
original_glrlm_ShortRunHighGrayLevelEmphasis	264.47±261.21	158.03±94.47	0.562
original_glrlm_ShortRunEmphasis	0.96±0.01	0.97±0.02	0.248
original_glrlm_RunVariance	0.05±0.02	0.05±0.03	0.4
original_glrlm_RunPercentage	0.95±0.02	0.96±0.02	0.065
original_glrlm_RunLengthNonUniformityNormalized	0.91±0.03	0.92±0.04	0.223
original_glrlm_RunLengthNonUniformity	54.71±29.03	50.01±49.90	0.562
original_glrlm_RunEntropy	4.27±0.54	3.91±0.45	0.001
original_glrlm_LowGrayLevelRunEmphasis	0.05±0.03	0.06±0.03	0.077
original_glrlm_LongRunLowGrayLevelEmphasis	0.05±0.03	0.06±0.03	0.065
original_glrlm_LongRunHighGrayLevelEmphasis	303.53±280.44	181.15±99.72	0.017
original_glrlm_LongRunEmphasis	1.16±0.06	1.15±0.08	0.335
original_glrlm_HighGrayLevelRunEmphasis	271.87±264.80	162.39±95.28	0.024
original_glrlm_GrayLevelVariance	55.40±68.97	27.96±16.10	0.027
original_glrlm_GrayLevelNonUniformityNormalized	0.07±0.03	0.09±0.02	0.004
original_glrlm_GrayLevelNonUniformity	3.70±1.55	4.13±3.16	0.374
original_gldm_SmallDependenceLowGrayLevelEmphasis	0.04±0.02	0.05±0.03	0.112
original_gldm_SmallDependenceHighGrayLevelEmphasis	219.85±237.21	130.48±87.23	0.04
original_gldm_SmallDependenceEmphasis	0.76±0.07	0.76±0.11	0.608
original_gldm_LowGrayLevelEmphasis	0.04±0.02	0.05±0.03	0.084
original_gldm_LargeDependenceLowGrayLevelEmphasis	0.07±0.04	0.08±0.05	0.073
original_gldm_LargeDependenceHighGrayLevelEmphasis	526.95±410.30	344.18±267.85	0.023
original_gldm_LargeDependenceEmphasis	2.31±0.56	2.28±0.89	0.855
original_gldm_HighGrayLevelEmphasis	270.22±263.11	161.15±94.07	0.024
original_gldm_GrayLevelVariance	54.60±68.51	27.56±16.00	0.028
original_gldm_GrayLevelNonUniformityNormalized	0.071±0.026	0.087±0.025	0.006

original_gldm_GrayLevelNonUniformity	3.98±1.72	4.44±3.44	0.39
original_gldm_DependenceVariance	0.37±0.19	0.36±0.26	0.738
original_gldm_DependenceNonUniformityNormalized	0.55±0.10	0.58±0.16	0.332
original_gldm_DependenceNonUniformity	34.72±18.96	28.07±18.20	0.102
original_gldm_DependenceEntropy	4.62±0.55	4.27±0.68	0.007
original_glcm_SumSquares	55.03±71.17	28.54±17.45	0.038
original_glcm_SumEntropy	4.35±0.68	3.77±0.87	0.001
original_glcm_SumAverage	28.07±11.91	22.84±6.28	0.021
original_glcm_MaximumProbability	0.04±0.02	0.06±0.05	0.004
original_glcm_JointEnergy	0.02±0.01	0.04±0.05	0.006
original_glcm_JointAverage	14.03±5.96	11.42±3.14	0.021
original_glcm_InverseVariance	0.19±0.05	0.19±0.06	0.751
original_glcm_Imc2	0.99±0.01	0.98±0.16	0.016
original_glcm_Imc1	0.52±0.11	0.53±0.16	0.734
original_glcm_Idn	0.85±0.03	0.82±0.05	0.002
original_glcm_Idmn	0.95±0.02	0.92±0.05	0.001
original_glcm_Idm	0.18±0.04	0.18±0.05	0.756
original_glcm_Id	0.27±0.04	0.27±0.05	0.854
original_glcm_Homogeneity2	0.18±0.04	0.18±0.05	0.756
original_glcm_Homogeneity1	0.27±0.07	0.27±0.05	0.854
original_glcm_Entropy	6.01±0.84	5.43±1.21	0.007
original_glcm_Dissimilarity	5.72±2.20	5.39±1.79	0.455
original_glcm_DifferenceVariance	26.18±29.94	17.17±16.07	0.112
original_glcm_DifferenceEntropy	3.15±0.36	2.98±0.25	0.003
original_glcm_DifferenceAverage	5.72±2.20	5.39±1.79	0.455
original_glcm_Correlation	0.28±0.19	0.13±0.25	0.001
original_glcm_Contrast	64.42±62.57	51.01±40.39	0.269
original_glcm_ClusterTendency	155.72±228.47	63.16±37.16	0.023
original_glcm_ClusterShade	1403.74±593.07	154.87±384.81	0.129
original_glcm_ClusterProminence	201614.94±616786.94	17293.63±19968.35	0.09
original_glcm_Autocorrelation	254.79±266.19	143.11±86.91	0.021
original_firstorder_Variance	34067.01±42704.46	17132.30±9885.43	0.027
original_firstorder_Uniformity	0.071±0.026	0.087±0.025	0.006
original_firstorder_TotalEnergy	12037569.8±14797063.1	6279463.22±4797646.78	0.032
original_firstorder_StandardDeviation	164.06±33.66	126.62±33.66	0.017
original_firstorder_Skewness	0.36±0.48	0.32±0.68	0.689
original_firstorder_RootMeanSquared	492.46±469.69	456.79±121.44	0.288
original_firstorder_RobustMeanAbsoluteDeviation	90.23±50.05	68.20±17.83	0.016
original_firstorder_Range	749.69±381.58	566.09±177.55	0.01
original_firstorder_Minimum	146.11±92.13	183.36±126.72	0.104
original_firstorder_Median	444.33±141.13	430.33±121.45	0.631
original_firstorder_MeanAbsoluteDeviation	130.91±69.66	100.53±27.06	0.018
original_firstorder_Mean	462.22±153.36	437.28±122.75	0.422
original_firstorder_Maximum	895.51±386.99	749.45±183.47	0.043
original_firstorder_Kurtosis	3.10±0.86	3.42±2.64	0.389

original_firstorder_InterquartileRange	215.73±125.02	164.16±53.33	0.026
original_firstorder_Entropy	4.12±0.56	3.78±0.39	0.002
original_firstorder_Energy	19260047.2±23675231.8	11358732.6±10602419.9	0.072
original_firstorder_90Percentile	678.39±260.49	596.45±143.59	0.097
original_firstorder_10Percentile	270.77±93.74	290.70±115.21	0.365
general_info_VoxelNum	63.27±34.07	58.39±58.37	0.608
