# Supplementary figures and images for: Identification of high-risk plaque features in intracranial atherosclerosis: initial experience using a radiomic approach
Source: Eur Radiol. 2018 Apr 9;28(9):3912–21. doi: 10.1007/s00330-018-5395-1 (PMC6081255; doi:10.1007/s00330-018-5395-1)

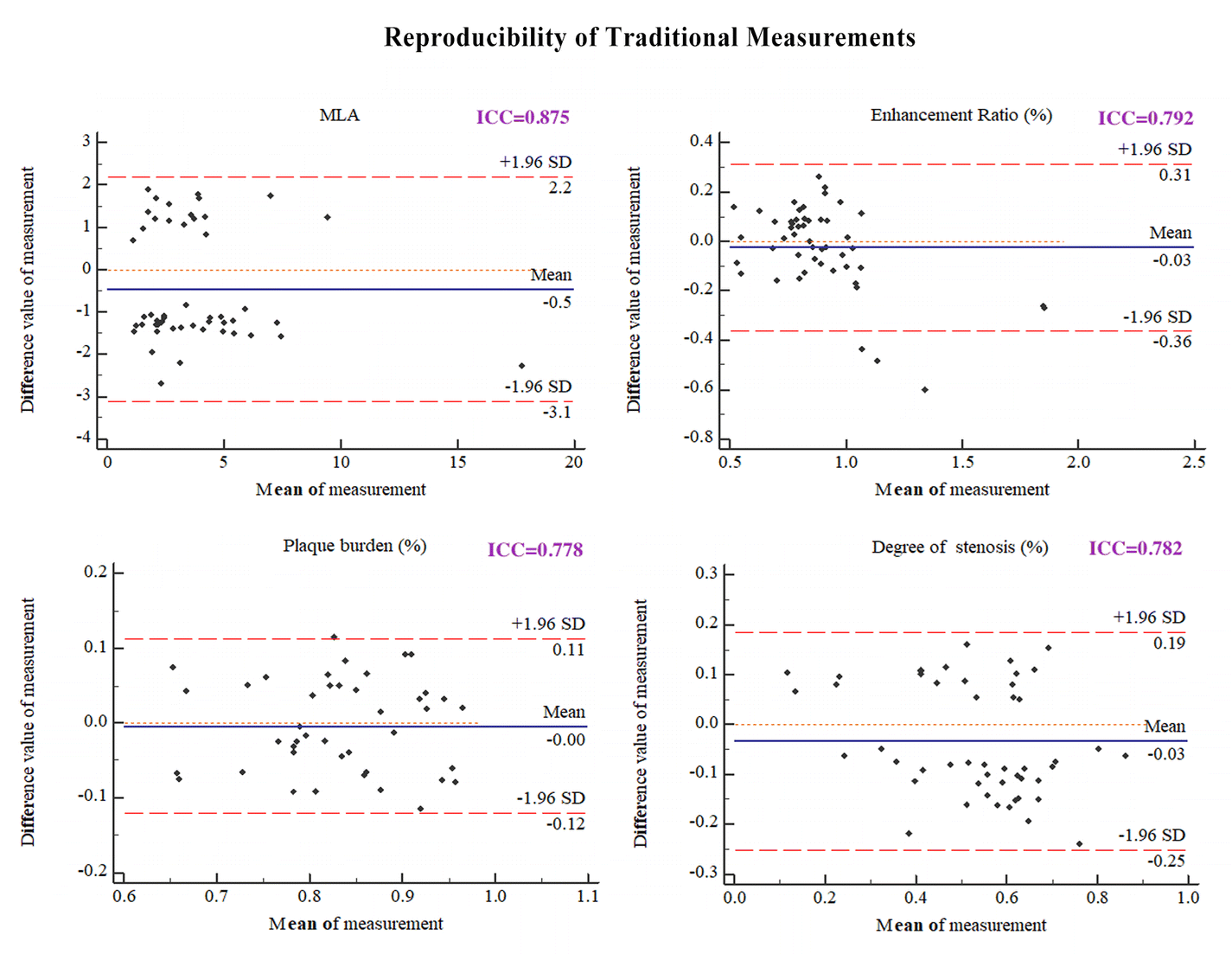

Supplement: Supplementary file 2 — (GIF 142 kb) [file 330_2018_5395_Fig4_ESM.gif]

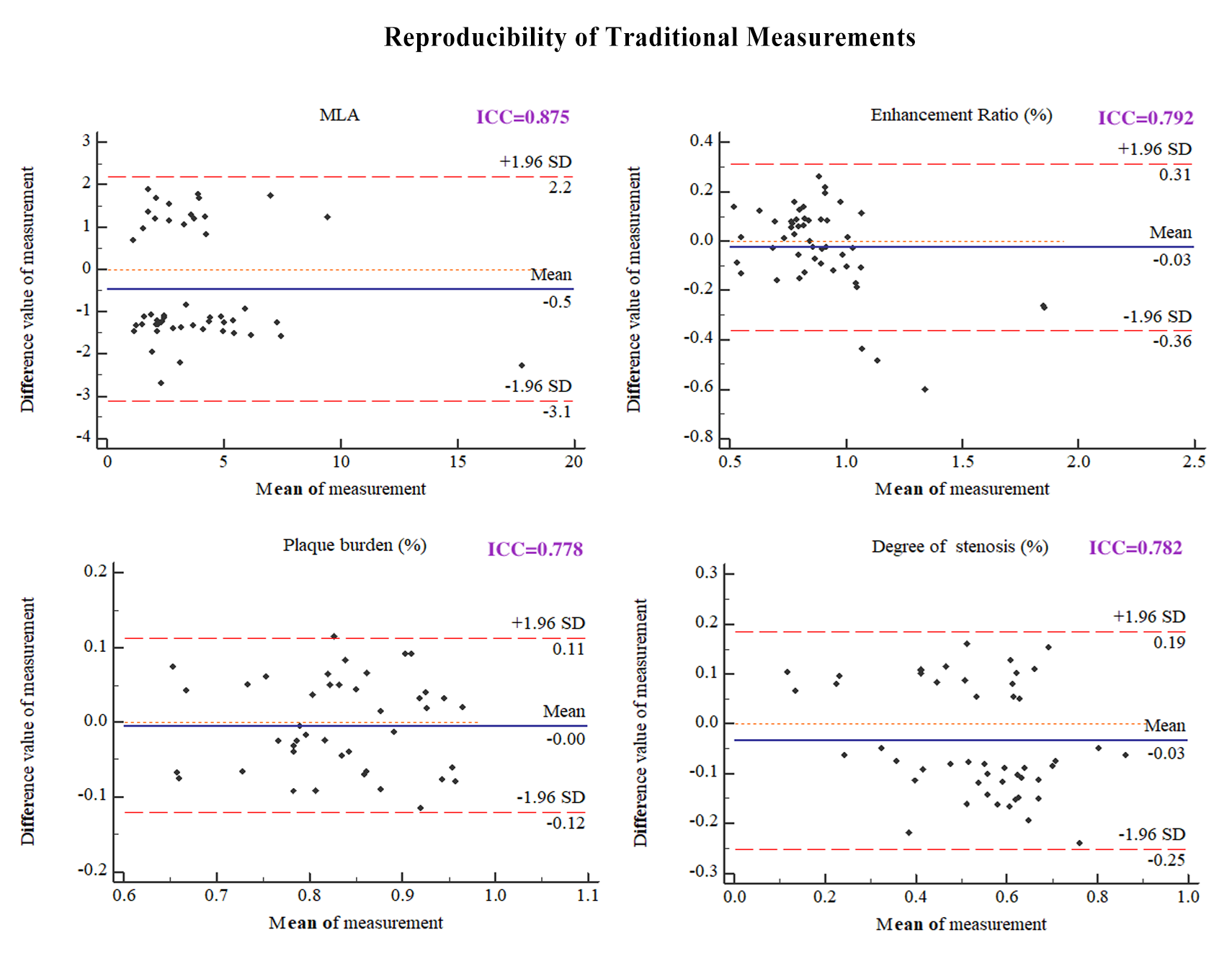

Supplement: Supplementary file 3 — High resolution image (TIFF 7376 kb) [file 330_2018_5395_MOESM2_ESM.tif]

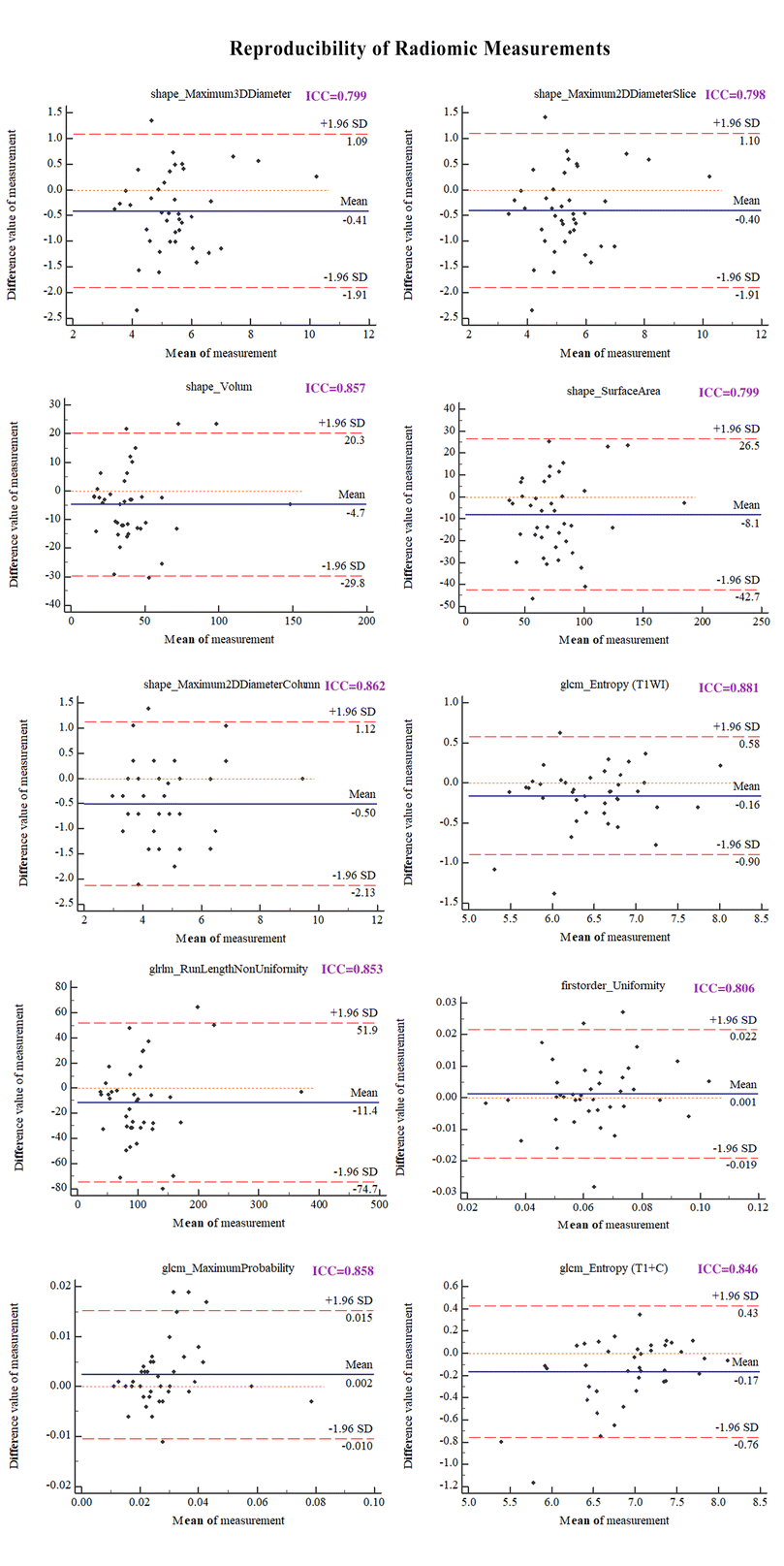

Supplement: Supplementary file 4 — (GIF 134 kb) [file 330_2018_5395_Fig5_ESM.gif]

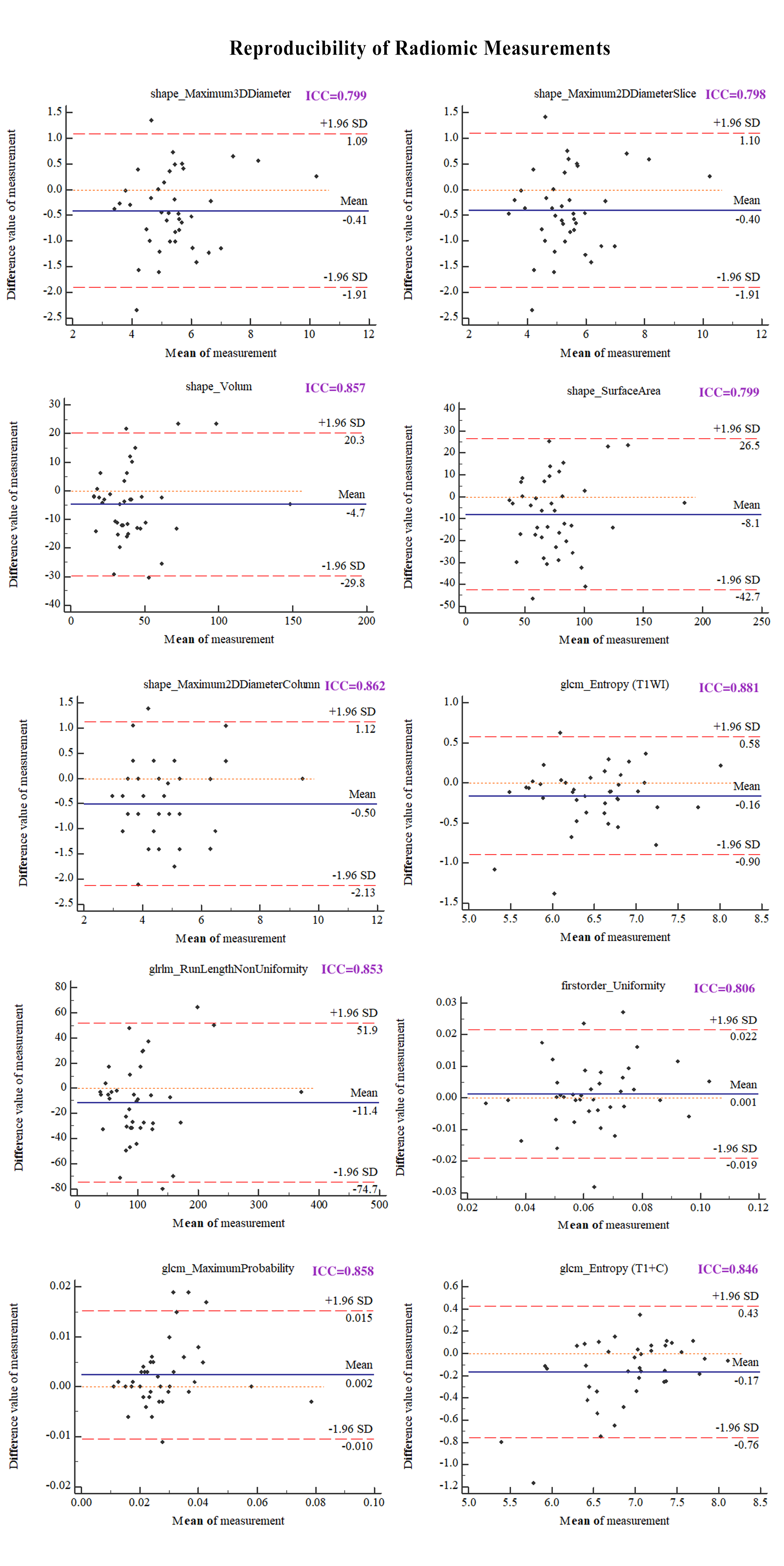

Supplement: Supplementary file 5 — High resolution image (TIFF 26585 kb) [file 330_2018_5395_MOESM3_ESM.tif]
